# Is There a Best First Line Biological/Small Molecule in IBD: Are We Ready for Sequencing?

**DOI:** 10.3390/biomedicines10040749

**Published:** 2022-03-23

**Authors:** Gustavo Drügg Hahn, Petra Anna Golovics, Panu Wetwittayakhlang, Alex Al Khoury, Talat Bessissow, Peter Laszlo Lakatos

**Affiliations:** 1 Division of Gastroenterology, McGill University Health Centre, Montreal, QC H3G 1A4, Canada; golovics.petra@gmail.com (P.A.G.); wet.panu@gmail.com (P.W.); talat.bessissow@gmail.com (T.B.); 2 School of Medicine, Graduate Course Sciences in Gastroenterology and Hepatology, Universidade Federal do Rio Grande do Sul, Porto Alegre 90035-002, Brazil; 3 Department of Gastroenterology, Hungarian Defence Forces, Medical Centre, H-1062 Budapest, Hungary; 4 Unit of Gastroenterology and Hepatology, Division of Internal Medicine, Faculty of Medicine, Prince of Songkla University, Songkhla 90110, Thailand; 5 Division of Gastroenterology, University of Florida Jacksonville, Jacksonville, FL 32209, USA; alexkhoury88@gmail.com; 6 1st Department of Medicine, Semmelweis University, H-1083 Budapest, Hungary

**Keywords:** inflammatory bowel disease, biologic therapy, small molecule, biologic-naïve

## Abstract

Inflammatory bowel disease (IBD) is a chronic, life-long inflammatory condition of the gastrointestinal tract. Treatment strategy depends on the severity of the disease course. IBD physicians need to be aware of the life-long treatment options available. The goal is not only to achieve clinical remission but to halt or stabilize the chronic inflammation in the intestines to prevent further structural damage. Therefore, the use of early biologic therapy is recommended in moderate-to-severe IBD patients. However, in the last decade, use of therapeutic drug monitoring has increased considerably, opening an opportunity for sequencing. This review summarizes the available evidence on biologic and small molecules therapy in Crohn’s disease (CD) and ulcerative colitis (UC) in different clinical scenarios, including perianal CD, the elderly, extra intestinal manifestations, and pregnancy.

## 1. Introduction

Biologic therapies have revolutionized the medical management of inflammatory bowel disease (IBD) in the last two decades and are associated with improved outcomes. However, individualized therapy should be considered for each patient, especially in those with different disease spectrums. The availability of biologic therapy opens an opportunity for sequencing. Nevertheless, high variability of individual factors, lack of data from head-to-head trials, and limited generalization of clinical trial results make selections difficult [1,2].

There are still insufficient data regarding optimal treatment and follow-up strategies in IBD. Therefore, the aim of this article is to review the available evidence on biologic and small molecules therapy in both CD and UC in different clinical scenarios, including unique situations such as perianal CD, elderly patients, extra intestinal manifestations, and pregnancy. In this manner, we hope to shed light on this for physicians, helping them with the decision process in their routine IBD care.

## 2. Evidence for Efficacy of Biologic Therapies in Naïve Patients from the Pivotal Clinical Trials

The first biologic therapy groups were the anti-TNFs, including infliximab (IFX) and adalimumab (ADA). Most of the available data are on these two drugs. We will focus on biologic-naïve patients to help guide physicians regarding the optimal first line therapy.

### 2.1. Anti-TNFs

The ACCENT I trial [3] aimed to evaluate IFX as a maintenance therapy for CD. Fifty-eight percent of patients responded to a single infusion of infliximab within 2 weeks, and at week 30, 21% of group I patients were in remission, compared with 39% of group II (*p* = 0.003) and 45% of group III (*p* = 0.0002) patients. Thus, patients in groups II and III combined were more likely to sustain clinical remission than patients in group I (odds ratio 2.7, 95% CI 1.6–4.6). Thus, this study concluded that patients with CD are more likely to be in remission at weeks 30 and 54, to be free of steroids, and to maintain their response for a longer period of time when maintained at every 8 weeks.

Regarding IFX as an induction and maintenance therapy for UC there are the ACT I and II trials [4]. In ACT I, 69% patients who received 5 mg of IFX and 61% who received 10 mg had a clinical response at week 8, as compared with 37% who received placebo (*p* < 0.001 for both comparisons with placebo). In ACT I, more patients who received 5 mg or 10 mg of IFX had a clinical response at week 54 than did those who received placebo (*p* < 0.001 for both comparisons). In ACT II, 64% of patients who received 5 mg of IFX and 69% who received 10 mg had a clinical response at week 8, as compared with 29% who received placebo (*p* < 0.001 for both comparisons with placebo). Both studies reported that patients who received IFX were more likely to have a clinical response at week 30 (*p* ≤ 0.002 for all comparisons). In conclusion, patients with moderate-to-severe active UC treated with IFX at induction phase (weeks 0, 2, and 6) and at the maintenance phase at every eight weeks were more likely to have a clinical response at weeks 8, 30, and 54 compared to placebo.

Regarding Adalimumab (ADA), the CHARM Trial [5] evaluated its efficacy and safety in maintenance of response and remission in patients with moderate-to-severe CD. The percentage of randomized responders in remission was significantly greater in the ADA 40 mg every 2 weeks and 40 mg weekly groups versus placebo at week 26 (*p* < 0.001) and week 56 (*p* < 0.001). No significant differences in efficacy between ADA regimens were observed. In addition, a higher number of patients on placebo discontinued treatment because of an adverse event than those on ADA. Therefore, the study concluded that amongst patients who responded to ADA, both regimens were significantly more effective than placebo in maintaining remission in moderate-to-severe CD through 56 weeks. ADA was well-tolerated with a consistent safety profile.

ULTRA 2 trial [6] evaluated the efficacy of ADA as a maintenance therapy for patients with UC. As main result, rates of clinical remission at week 8 were 16.5% on ADA and 9.3% on placebo (*p* = 0.019); values for week 52 were 17.3% and 8.5%, respectively (*p* = 0.004). Among anti-TNF-α naïve patients, rates of remission at week 8 were 21.3% on ADA and 11% on placebo (*p* = 0.017); values for week 52 were 22% and 12.4% (*p* = 0.029). The study concluded that ADA was safe and more effective than placebo as an induction and maintenance therapy to achieve clinical remission in patients with moderate-to-severe ulcerative colitis who did not have an adequate response to conventional therapy with steroids or immunosuppressants.

SERENE CD trial [7] aimed to evaluate higher versus standard ADA induction dosing and clinically adjusted versus therapeutic drug monitoring (TDM) maintenance strategies in patients with moderately to severely active CD. The study showed similar proportions of patients receiving higher induction dosing and standard induction dosing achieved clinical remission at week 4 (44% in both; *p* = 0.939) and endoscopic response at week 12 (43% vs. 39%, respectively, *p* = 0.462). Efficacy at week 56 was similar between clinically adjusted and TDM groups.

On the other hand, SERENE UC trial [8] evaluated the efficacy of a higher ADA induction and maintenance dose regimens in patients with UC. In this trial, 13.3% vs. 10.9% of patients on high induction regime versus standard achieved clinical remission at week 8 (*p* = 0.265), respectively. Amongst week-8 responders, 39.5% vs. 29.0% on 40 mg once a week versus 40 mg every 2 weeks achieved clinical remission at week 52 (*p* = 0.069), respectively. Safety profiles were comparable between the groups. Although primary endpoints were not met, an absolute difference over 10% in clinical remission was demonstrated with higher ADA regimen. In conclusion, higher dosing regimens were well-tolerated and consistent with the known safety profile of ADA in UC.

### 2.2. Combination Therapy—Anti-TNF + Immunomodulator

In the SONIC trial [9], the primary endpoint was steroid-free clinical remission in patients treated with IFX and azathioprine (AZA) mono or combination therapy. There was a significant difference in favor of the combination therapy.

An early single center real-life study from Leuven showed that IFX is effective treatment for a CD patient and maintained improvement and decreased the hospitalization and surgery rate during the observation period. [10]

The SUCCESS study [11] was designed for combination therapy in UC. Results showed that, at week 16, combination therapy with IFX and AZA showed significant steroid-free remission rate (*p* = 0.017 and *p* = 0.032) compared to either monotherapy and led to a significantly better mucosal healing.

### 2.3. Vedolizumab

GEMINI I trial [12] evaluated vedolizumab (VDZ) as a potential therapy for induction and maintenance in patients with UC. The study showed response rates at week 6 to be 47.1% and 25.5% among patients in the VDZ group and placebo group, respectively (*p* < 0.001). At week 52, 41.8% of patients who continued to receive VDZ every 8 weeks and 44.8% of patients who continued to receive VDZ every 4 weeks were in clinical remission, as compared to 15.9% of patients who were switched to placebo (*p* < 0.001). Frequency of adverse events was similar in the vedolizumab and placebo groups. In conclusion, VDZ was more effective than placebo as an induction and maintenance therapy for UC.

GEMINI II trial [13] evaluated VDZ as an induction and maintenance therapy for CD. At week 6, a total of 14.5% of the patients in cohort 1 who received VDZ and 6.8% who received placebo were in clinical remission (*p* = 0.02). Amongst patients in cohorts 1 and 2 who had a response to induction therapy, 39% and 36.4% on VDZ every 8 weeks and every 4 weeks, respectively, were in clinical remission at week 52, as compared to 21.6% on placebo (*p* < 0.001 and *p* = 0.004 for the two VDZ groups, respectively, vs. placebo). VDZ was associated with a higher rate of serious adverse events (24.4% vs. 15.3%), infections (44.1% vs. 40.2%), and serious infections (5.5% vs. 3.0%), compared to placebo. Thus, this trial concluded that patients with active CD treated with VDZ were more likely to be in remission than patients on placebo. In addition, patients with a response to induction therapy who continued to receive VDZ were more likely to be in remission at week 52.

### 2.4. Ustekinumab

IM-UNITI trial [14] studied UST as induction and maintenance therapy for CD. The study reported significantly higher rates of response at week 6 amongst patients on intravenous UST at a dose of either 130 mg or approximately 6 mg per kilogram was significantly higher when compared to patients on placebo (*p* ≤ 0.003). In the maintenance dose groups of UST every 8 weeks or every 12 weeks, 53.1% and 48.8%, respectively, were in remission at week 44, as compared with 35.9% of those on placebo (*p* = 0.005 and *p* = 0.04, respectively). In conclusion, a significantly higher rate of response was seen among patients with moderately to severely active CD receiving intravenous UST compared to placebo. In addition, subcutaneous UST maintained remission in patients who had a clinical response to induction therapy.

UNIFI trial [15] evaluated UST as an induction and maintenance therapy in patients with UC. It reported a significantly higher rate of clinical remission at week 8 among patients who received intravenous UST at a dose of 130 mg (15.6%) or 6 mg per kilogram (15.5%) compared to patients who received placebo (5.3%) (*p* < 0.001 for both comparisons). Among patients who had a response to induction therapy with UST and underwent a second randomization, the percentage of patients who had clinical remission at week 44 was significantly higher among patients assigned to 90 mg of subcutaneous UST every 12 weeks (38.4%) or every 8 weeks (43.8%) than among those on placebo (24.0%) (*p* = 0.002 and *p* < 0.001, respectively). Therefore, the study concluded UST to be more effective in inducing and maintaining remission in patients with moderate-to-severe UC compared to placebo.

### 2.5. Risankizumab

Risankizumab (RZB), a selective interleukin-23 inhibitor, demonstrated superior efficacy over placebo (PBO) as an induction and maintenance therapy in patients with moderate-to-severe CD. ADVANCE and MOTIVATE studies [16] evaluated RZB as an induction therapy in patients with moderate-to-severe CD. In both studies, starting at week 4, greater proportions of RZB 600 mg or RZB 1200 mg versus PBO-treated patients achieved clinical remission per either CDAI (*p* = 0.01/*p* < 0.05) or SF/AP criteria (*p* < 0.01/*p* < 0.01), clinical response per CDAI criterion (*p* = 0.001/*p* < 0.01) and enhanced clinical response per SF/AP criteria (*p* < 0.01/*p* = 0.14). The efficacy and treatment effect increased through week 12 (*p* ≤ 0.001/*p* ≤ 0.001) for both RZB doses. Treatment with RZB 600 mg or 1200 mg was well-tolerated, and no new safety risks were identified. These studies concluded that induction therapy with both RZB 600 mg and 1200 mg intravenous resulted in significantly greater clinical remission and response compared to PBO as early as week 4 and sustained through week 12 in patients with moderate-to-severe CD who had inadequate response or intolerance to conventional and/or biologic treatment.

FORTIFY study [17] evaluated the effectiveness of RZB as a maintenance therapy in patients with moderate-to-severe CD. For the main results, at 12 weeks of IV RZB induction therapy, 141 patients were randomized to RZB 360 mg (patients achieving endoscopic response, 55/141; endoscopic remission, 39/141; SES-CD score of 0–2, 29/141) and 164 were randomized to withdrawal (placebo SC) (patients achieving endoscopic response, 73/164; endoscopic remission, 46/164; SES-CD 0–2, 32/164). Maintenance of endoscopic response at week 52 was demonstrated in 70.2% of patients on RZB 360 mg SC versus 38.4% of patients in the placebo arm (*p* < 0.001). Maintenance of endoscopic remission at week 52 was demonstrated in 74.4% of patients receiving RZB 360 mg versus 23.9% of patients in the placebo arm (*p* < 0.001). RZB maintenance treatment was well-tolerated and no new safety signals were observed. In conclusion, this study showed that RZB IV induction followed by SC maintenance therapy led to sustained improvements in endoscopic outcomes, demonstrating the durability of efficacy with continued RZB treatment in patients with moderate-to-severe CD.

### 2.6. Tofacitinib

OCTAVE trials [18] evaluated Tofacitinib (TOFA) as an induction and maintenance therapy for UC. The study reported remission at week 8 in 18.5% of the patients in the TOFA group versus 8.2% in the placebo group (*p* = 0.007); in the OCTAVE Induction 2 trial, remission occurred in 16.6% versus 3.6% (*p* < 0.001). In the OCTAVE Sustain trial, remission at 52 weeks occurred in 34.3% of the patients receiving 5 mg TOFA and 40.6% receiving 10 mg TOFA versus 11.1% in the placebo group (*p* < 0.001 for both comparisons with placebo). As a conclusion, TOFA was more effective as an induction and maintenance therapy compared to placebo in patients with moderate-to-severe active UC.

### 2.7. Upadacitinib

Upadacitinib (UPA), a more selective oral JAK inhibitor, showed significantly greater efficacy compared to placebo (PBO) in induction treatment of patients with moderately to severely active UC in two phase 3 induction trials, U-ACHIEVE and U-ACCOMPLISH [19]. In both studies, approximately half the patients had previously failed a biologic therapy. In both groups (non-biologic failure versus bio-failed), a significantly higher proportion of patients on UPA achieved clinical remission versus placebo; the magnitude of clinical remission at week 8 was greater in non-biologic failure patients (UPA, 35% vs. PBO, 9%; treatment difference [95% CI]: 26.0% [16.0, 36.1]) versus bio-failed (UPA, 18% vs. PBO, 0%; 17.5% [11.4, 23.6]) in U-ACHIEVE and non-bio-failed (UPA, 38% vs. PBO, 6%; 31.6% [22.8, 40.5]) versus bio-failed (UPA, 30% vs. PBO, 2%; 27.1% [19.6, 34.7]) in U-ACCOMPLISH. UPA 45 mg QD was well-tolerated and no new safety signals were observed. In summary, UPA 45 mg QD was demonstrated to be an effective induction treatment for patients with moderately to severely active UC.

Regarding UPA as a maintenance therapy in UC, Panaccione et al. [20] reported that in patients responding to UPA induction therapy both UPA 15 mg and UPA 30 mg were safe and effective as maintenance treatment at 52 weeks for all primary and secondary endpoints (*p* < 0.001 for all endpoints). Patients on UPA 30 mg responded approximately 10% better for most endpoints compared to those on UPA 15 mg. Both doses were well-tolerated, with no new safety signals observed.

### 2.8. Ozanimod

Ozanimod (OZN) represents a selective sphingosine-1-phosphate receptor modulator originally used in multiple sclerosis and now has been recently approved for the treatment of UC [21]. The incidence of clinical remission was significantly higher among patients on OZN than among those on PBO during both induction (18.4% vs. 6.0%, *p* < 0.001) and maintenance (37.0% vs. 18.5% [at week 10], *p* < 0.001). The incidence of clinical response was also significantly higher with OZN than with PBO during induction (47.8% vs. 25.9%, *p* < 0.001) and maintenance (60.0% vs. 41.0%, *p* < 0.001). The incidence of infection (of any severity) with OZN was similar to PBO during induction and higher than that of PBO during maintenance. Serious infection occurred in less than 2% of the patients in each group during the 52 weeks. Elevated liver aminotransferase levels were more common with OZN. In conclusion, OZN was more effective compared to PBO as induction and maintenance therapy in patients with moderately to severely active UC.

Moreover, OZN may be associated with bradycardia and atrioventricular conduction delays. In a phase 3 UC trial [22], continuous OZN treatment was not associated with any clinically significant changes in heart rate or electrocardiogram. The incidence of cardiac-related treatment-emergent adverse events with OZN during induction in Cohorts 1 and 2 was low. This study concluded that OZN had a manageable long-term cardiac safety profile with a low incidence of bradycardia and few serious long-term cardiac safety findings.

OZN represents a new and promising treatment; however, further data are needed for the safety concerns of cardiac adverse events and ophthalmology complications.

## 3. Head-to-Head Clinical Trials

This section will summarize the few head-to-head trials that compared effectiveness of the available biological therapies.

The VARSITY study [23] compared the effectiveness of intravenous (IV) VDZ and subcutaneous (SC) VDZ versus ADA in moderate-to-severe UC patients. The previous exposure to a tumor necrosis factor inhibitor other than adalimumab was allowed in up to 25% of patients. Dose escalation was not permitted in either group. There was a total of 769 patients (ratio 1:1) at week 52, clinical remission was observed in a higher percentage of patients in the vedolizumab group than in the adalimumab group (31.3% vs. 22.5%; 95% CI:2.5 to 15.0; *p* = 0.006), as was endoscopic improvement (39.7% vs. 27.7%; 95% CI, 5.3 to 18.5; *p* < 0.001). Corticosteroid-free clinical remission occurred in 12.6% of the patients in the vedolizumab group and in 21.8% in the adalimumab group (95% CI, −18.9 to 0.4). Exposure-adjusted incidence rates of infection were 23.4 and 34.6 events per 100 patient-years with vedolizumab and adalimumab, respectively, and the corresponding rates for serious infection were 1.6 and 2.2 events per 100 patient-years. In conclusion, vedolizumab was superior to adalimumab with respect to achievement of clinical remission and endoscopic improvement, but not corticosteroid-free clinical remission.

The SEAVUE study [24] compared the efficacy and the safety of UST versus ADA for induction and maintenance therapy in moderate-to-severe Crohn’s disease. In all, 386 patients were randomized (ratio 1:1) and at week 52, 65% of UST and 61% of ADA patients achieved clinical remission (*p* = 0.417). The endoscopic remission and the corticosteroid free remission were similar between the two groups. The infection rates were 34% in the UST-treated group and 40.4% in the ADA-treated group and SAE was 2.6% vs. 7.2%; however, these data are not statistically significant.

## 4. Real-World Studies of Biological Therapies in Crohn’s Disease

Multiple real-world studies compared the efficacy and safety between different biologics in CD. Most existing studies compared infliximab with adalimumab as the first biologic therapy in CD. Few real-world studies compared anti-TNFs with vedolizumab or ustekinumab in biologic-naïve patients with CD, given these new biologic therapies are generally used in treating patients with anti-TNF refractory Crohn’s disease.

Infliximab and adalimumab have shown to have comparable efficacy for the treatment of CD in biologic-naïve patients in multiple studies [25,26,27,28,29,30,31]. In a retrospective of 1284 patients, 70% were biologic-naïve, clinical response rates at 6 and 24 months were comparable between patients treated with IFX and ADA (IFX 72% and 45% vs. ADA 64% and 44%, respectively) [27]. Narula et al. reported 362 patients with CD had similar rates of clinical remission (IFX 50.4% vs. ADA 57.3%) and steroid-free remission (IFX 44.3% vs. ADA 53.7%) at 12 months of treatment. Moreover, there is no difference in CD-related hospitalization, abdominal surgery, and serious infections between IFX and ADA therapy [26].

In comparing vedolizumab and anti-TNF therapy, a retrospective study comparing the effectiveness of VDZ with ADA included 585 patients with CD, and 57% were biologic-naïve patients. There was no difference between clinical response (54.0% VDZ vs. 69.1% ADA, *p* = 0.33) and endoscopic healing (31.8% VDZ vs. 33.8% ADA, *p* = 0.85) at week 52 between the groups [31].

However, another cohort showed that VDZ was favored in achieving clinical remission (HR 1.861, 95% CI 1.06–3.27) and steroid-free remission (HR 5.60, 95% CI 1.47–21.37) over subcutaneous anti-TNF, but not significantly different between VDZ and IFX. The rates of non-infectious SAEs (OR, 0.072, 95% CI 0.01–0.24) but not serious infections (OR 1.183, 95% CI 0.78–1.79) were significantly lower with VDZ than anti-TNFs [32].

A recent real-world assessing treatment persistence at 12 months demonstrated that ustekinumab (80%) remained the highest as first-line therapy, followed by VDZ 73.5%, IFX 68.1%, and ADA 64.2% (*p* = 0.01), respectively [32].

The summary of selected studies comparing two biologics in biologic-naïve patients with Crohn’s disease is shown in Table 1.

## 5. Real-World Studies of Biological Therapies in Ulcerative Colitis

Multiple real-world studies assessed the efficacy of biologic therapies and small molecules in UC. However, the number of studies comparing two therapies is relatively limited in biologic-naïve patients (Table 2).

In a nationwide register-based study of 275 biologic-naïve patients with UC who were treated with IFX or ADA, adalimumab was associated with a higher risk of hospitalization (HR, 1.84) and serious infections (HR, 5.11) compared to patients treated with IFX. However, the risk of abdominal surgery was not different between the groups [34].

In a large retrospective study of 604 biologic-naïve patients with UC, Bressler et al. reported equal efficacy in patients treated with vedolizumab and anti-TNFs with regards to clinical remission (VDZ 65.9% vs. anti-TNF 48.6%; *p* = 0.09) and mucosal healing (VDZ 86.6% vs. anti-TNF 80.6%; *p* = 0.66;) over 24 months. However, incidence rates of disease exacerbations were lower in vedolizumab patients (HR = 0.58, 95% CI 0.45–0.76). The vedolizumab patients were less likely to experience SAEs (HR = 0.37, 95% CI 0.21–0.63), but not serious infections (HR = 0.56, 95% CI 0.21–1.51) [35]. In addition, several small retrospective studies have shown comparable rates of clinical response and remission between VDZ and anti-TNFs in biologic-naïve patients with UC [36,37,38].

Vedolizumab has been shown to have higher efficacy in a recent multi-center observational cohort in 303 biologic-naïve patients when compared to anti-TNFs. The patients treated with vedolizumab achieved higher clinical remission (HR, 1.67; 95% CI, 1.15–2.43) and deep remission (HR, 5.24; 95% CI, 1.18–23.19) than those treated with anti-TNF [39]. The results were consistent in a subgroup analysis of infliximab and adalimumab. Similar safety results with previous study, vedolizumab was associated with a significantly lower risk for SAEs, but nonsignificant trends toward lower risk for serious infection.

In a retrospective cohort comparing IFX with VDZ, treatment persistence rates were higher for VDZ versus IFX at 24 months post-maintenance therapy (VDZ 78.5% vs. IFX 63.5%, *p* = 0.046). Similarly, another study showed that vedolizumab had a higher rate of treatment persistence as a first-line biologic in UC (VDZ 73.4% vs. IFX 61.1% vs. ADA 45.5%, *p* < 0.001) [33,40].

**Table 2:** Summary of selected real-world studies comparing biologics in biologic-naïve patients with UC.

Study (Year)	Biologic Therapy	Nature of the Study	Studied Population	No. of Biologic-Naïve Patients	Main Result
Singh et al. (2017) [35]	IFX vs. ADA	Multicentric Retrospective Cohort	Caucasian Population	IFX (171) ADA (104)	ADA vs. IFX, UC-related hospitalization (HR, 1.71; 95% CI, 0.95–3.07, all-cause hospitalization (HR 1.84; 95% CI, 1.18–2.85) and serious infections (HR, 5.11; 95% CI, 1.20–21.80).
Patel et al. (2019) [40]	IFX vs. VDZ	Multicentric Retrospective Cohort	North American Population	IFX (469) VDZ (247)	Rates of treatment persistence were numerically higher at 3 and 12 months for VDZ vs. IFX, and significant difference at 24 months (VDZ 78.5% vs. IFX 63.5%, *p* = 0.046)
Helwig et al. (2020) [38]	Anti-TNFs vs. VDZ	Multicentric Retrospective Cohort	Caucasian Population	Anti-TNFs (40) VDZ (22)	Clinical remission at Week 26 was 50.1% for VDZ vs. 31.5% for anti-TNF (*p* = ns)
Lukin et al. (2022) [39]	Anti-TNF vs. VDZ	Multicentric Retrospective Cohort	North American Population	Anti-TNF (160; 114 IFX, 87 ADA, 16 GOL) 143 VDZ	Higher rates of clinical remission in VDZ treated patients (HR, 1.67; 95% CI, 1.157–2.428) and deep remission (HR, 5.244; 95% CI, 1.186–23.193) SAEs (HR, 0.192; 95% CI, 0.049–0.754), but nonsignificant trends toward lower risk for serious infection (HR, 0.320; 95% CI, 0.078–1.322).
Bressler et al. (2021) [34]	Anti-TNFs vs. VDZ	Multicentric Retrospective Cohort	Caucasian and North American Population	Anti-TNFs (224; 138 IFX, 62 ADA, 24 GOL) VDZ (380)	clinical response [VDZ 88.3% vs. anti-TNF 86.2%, *p* = 0.64], Clinical remission (VDZ 65.9% vs. anti-TNF 48.6%; *p* = 0.09), and mucosal healing (VDZ 86.6% vs. anti-TNF 80.6%; *p* = 0.66); over 24 months.
Ko et al. (2021) [33]	Anti-TNFs vs. VDZ	Multicentric Retrospective Cohort	Australian Population	IFX (399) ADA (66) VDZ (167)	Rates of treatment persistence at 12-months, VDZ 73.4%, IFX 61.1% and ADA 45.5% (*p* < 0.001).

## 6. Network Meta-Analysis Comparing Biological and/or Small Molecule Therapies in Biologic-Naïve Patients

Only few studies compared head-to-head biologic therapy as a first-line treatment in patients with Crohn’s disease and UC. Therefore, network meta-analyses were developed to compare the efficacy of different biologic agents and small molecules therapies in biologic-naïve patients.

### 6.1. Network Meta-Analysis in Luminal Crohn’s Disease

A network meta-analysis was performed by Singh et al. using the surface under the cumulative ranking (SUCRA) probabilities as an indirect comparison to rank the efficacy of biologic agents in treating biologic-naïve patients with CD.

Infliximab (SUCRA 0.93) and adalimumab (SUCRA 0.75) were ranked the highest for induction and maintenance of clinical remission [41].

In a more recent meta-analysis of 2931 biologic-naïve patients with Crohn’s disease, IFX monotherapy (OR 4.53), IFX combined with azathioprine (OR 7.49), ADA (OR 3.01), and UST (OR 2.63) were associated with significantly higher odds of inducing remission compared to certolizumab [42]. In addition, a combination of infliximab and azathioprine therapy was associated with higher odds of remission when compared to vedolizumab (OR 3.76). The result from this meta-analysis suggests that either infliximab with azathioprine or adalimumab might be preferred as first-line therapy in moderate-to-severe CD patients.

### 6.2. Ulcerative Colitis

An early meta-analysis in 2017 showed that IFX is superior to ADA (OR 2.10, 95% CI 1.21–3.64) in clinical remission and superior to ADA (OR 1.87, 95% CI 1.26–2.79) and golimumab in terms of mucosal healing (OR 1.75, 95% CI 1.13–2.73). There was no difference in efficacy between tofacitinib and biologics [43]. In the same year, a meta-analysis by Singh et al. demonstrated that infliximab and vedolizumab were ranked highest for induction of clinical remission and mucosal healing (OR 4.10; 95% CI 2.58–6.52, SUCRA, 0.85 and vedolizumab SUCRA, 0.82) [44].

In 2021, a recent network meta-analysis of 15 RCTs was performed, including 3747 biologic-naïve patients with moderate-to-severe UC. This analysis included four trials of IFX, four trials of ADA, two trials of GOL, three trials of VDZ, two trials of TOFA, one trial with UST and one trial comparing head-to-head ADA vs. VDZ. Infliximab was ranked highest for induction of clinical remission (OR vs. placebo, 4.07; 95% CI, 2.67–6.21; SUCRA, 0.95) and endoscopic improvement (SUCRA 0.95) [45].

In more recent studies, Jarath et al. showed that the efficacy of anti-TNF agents was similar to VDZ. Infliximab had greater efficacy for induction response (OR 1.63, 1.15–2.30) and remission (OR 1.67, 95% CI 1.16–2.42). Adalimumab had lower induction, maintenance of response, and remission rates when compared to VDZ (OR 0.62, 95% CI 0.45–0.86) in anti-TNF-naïve patients. Tofacitinib had the highest maintenance treatment efficacy but the highest infection risk [46]. A network meta-analysis of 23 RCTs showed infliximab (SUCRA 0.853) and ozanimod (0.847) ranked the highest for the induction of clinical remission. Ustekinumab ranked highest for the induction of endoscopic improvement in biologic-naïve patients (SUCRA 0.825) [47].

Thus, current network meta-analysis suggests that anti-TNF, especially infliximab, is the most preferred biologic therapy for biologic-naïve patients with moderate-to-severe Crohn’s disease and ulcerative colitis as a first-line treatment. Vedolizumab has shown comparable efficacy to infliximab in network meta-analysis in naïve patients with UC but not CD. It is important to note that the efficacy of treatment in most of the meta-analyses was analyzed based on the indirect comparison that used ranking (SUCRA), which should be interpreted with caution. Of note, there are no thresholds for clinically meaningful differences between SUCRA values among the different agents. SUCRA does not consider the magnitude of differences in effects between treatments.

## 7. Biological Therapy in Special Situations

### 7.1. Perianal Fistulizing Crohn’s Disease

A fistula is an aggressive phenotype of CD that affects approximately 30% of patients [48]. Fistulizing Crohn’s disease (FCD) is more difficult to treat than luminal disease. Nonetheless, RCTs with a primary endpoint of treating FCD are scarce. Therefore, the current evidence is based mainly on post hoc analysis of RCTs retrospective or observational studies.

Infliximab is the only biologic that has proven efficacy in randomized placebo-controlled trials as a specific primary indication for treating FCD [49,50]. In the first RCT of 94 patients with FCD, the overall response rate was 62% with IFX compared to 26% with placebo (*p* = 0.002) [49,50]. In the ACCENT II trial, 195 of 282 patients (67%) had initially responded to induction therapy at week 14. At 54 weeks, patients with maintenance infliximab had a higher complete response compared to placebo patients (36% vs. 19%; *p* = 0.009). This study suggests that only one-third of FCD patients achieved fistula healing [51]. In another retrospective study of 178 FCD patients treated with IFX, clinical and radiological remission was observed in 55% and 38% of patients [52].

The CHARM trial was the first RCT that showed positive results of adalimumab in treating FCD. Fistula closure was achieved in 30% of 117 FCD patients treated with ADA compared to 13% with placebo at week 26 and week 56 (ADA 33% vs. placebo 13%, *p* = 0.016) [5]. The complete fistula healing was sustained in 90% of patients for up to 2 years [52]. The CHOICE trial was an open-label, single-arm study where 673 patients who had failed infliximab therapy were enrolled and treated with adalimumab induction and maintenance therapy; 13% had enterocutaneous or perianal fistulas. At the time of their last visit, 34/88 (39%) patients achieved complete fistula healing (range 4–36 weeks) [53]. Despite the lack of head-to-head RCT comparing the efficacy of biologics in FCD, several real-world studies have shown no clinical difference between infliximab and adalimumab in (62.5%; ADA vs. 83.9; IFX, *p* = 0.09) [54,55]. Nevertheless, evidence of certolizumab in treating FCD is low compared to other anti-TNF agents based on negative results from two RCTs [56,57].

There is no RCT assessing fistula response as a primary endpoint for ustekinumab and vedolizumab. Post hoc pool analysis from RCTs investigating the efficacy of UST (UNITI-1, UNITI-2, and CERTIFI study) included 150 patients with FCD treated with ustekinumab. Complete fistula healing was 24.7% in patients treated with UST compared to 14.1% in placebo-treated patients (*p* = 0.073) at 8 weeks [58]. In a meta-analysis of nine studies with a total of 396 patients treated with UST on perianal FCD, the pooled fistula remissions were 17.7% and 16.7% at weeks 24 and 52, respectively [59].

The efficacy of vedolizumab in FCD has been investigated in a post hoc analysis of the GEMINI 2 trial, including 153 patients. A higher proportion of patients receiving VDZ achieved fistula closure compared to placebo at week 52 (31% vs. 11%, ARR 19.7%, 95% CI −8.9 to 46.2) [60]. In a recent meta-analysis of four studies that included 198 patients with active perianal FCD, 87% had failed anti-TNF therapy. VDZ treatment led to the healing of perianal fistulas in 28% of the patients [61].

Recently, filgotinib (FIL) is a once-daily, oral JAK-1 inhibitor. The efficacy of FIL for the treatment of perianal CD was evaluated in phase 2 RCT (DIVERGENCE 2 study) of 57 patients, 65% of the patients had failed to anti-TNF. The patients treated with FIL 200 mg achieved a numerically higher rate of fistula response (FIL 47.1% vs. placebo 25.0%) and remission (FIL 47.1% vs. placebo 16.7%) than the placebo group at week 24 [62].

### 7.2. Elderly Patients with IBD

Currently, biologic therapy for the elderly IBD patients follows overall the same algorithms as in younger IBD patients. Treating elderly patients with IBD may be challenging compared to younger patients, not only due to advanced age and increasing number of comorbidities but also due to polypharmacy and age-related changes in the pharmacokinetic properties of the therapy [63,64].

A recent meta-analysis by Hahn et al. [65] evaluated the safety profile of the available biological therapies in the elderly IBD population. The rates of adverse events (AE) and infections were not different according to the type of biologics (AE mean rate: 11.3 (CI 95% 9.9–12.7)/100 pts years; *p* = 0.11, infection mean rate: 9.5 (CI 95% 8.4–10.6)/100 pts years; *p* = 0.56) in elderly IBD patients with the use of anti-TNF, VDZ, or UST. In addition, infusion/injection reaction rates were more common in patients on anti-TNFs (mean rate: 2.51 (CI 95% 1.7–3.4/100 pts years; *p* = 0.02) and malignancy rates were higher in elderly patients on VDZ/UST (mean rate: 2.14 (CI 95% 1.6–2.8)/100 pts years; *p* = 0.01). This latter may represent, though, a selection bias phenomenon, namely that the treating physician may be more likely to start UST or VDZ in patients with a high risk for malignancy based on the beneficial safety profile of the new biologicals reported from the landmark clinical trials.

Two studies directly compared the efficacy and safety of anti-TNFs and VDZ. Adar et al. [66] reported no significant differences in the safety profile between the two biologicals. In all, 113 patients (86%) of the anti-TNF group (total of 131) were anti-TNF naïve, and 123 (94%) were VDZ naïve. As for the VDZ group (103 patients), 41 patients (40%) were not previously exposed to anti-TNF. Infections were observed in 20% of patients treated with anti-TNF and 17% of treated patients with VDZ after 1 year of follow-up (*p* = 0.54).

Pabla et al. [67] included a total of 212 patients (108 treated with VDZ and 104 with anti-TNF). In the VDZ group, 79 patients (73.2%) had previously failed anti-TNF therapy along 18 patients (17.3%) in the anti-TNF group. This study showed that there were no significant differences between both cohorts in terms of rates of serious infections, surgical intervention, or IBD-hospitalization-free survival (*p* = ns).

### 7.3. Extraintestinal Manifestations

Extraintestinal manifestations (EIMs) can modify the therapeutic choice. For patients with IBD and EIMs, TNF antagonists tend to be preferred by gastroenterologists, and the ECCO guidelines recommend the use of TNF antagonists for patients with CD with various EIMs [68]. A pooled analysis of 11 induction, maintenance, and open-label extension studies of ADA demonstrated that more than 50% of patients receiving ADA achieved resolution of any EIM and arthritis/arthralgia at 6 months and 1 year in a significantly greater proportion of ADA vs. placebo [68]. There is high-quality evidence for the effectiveness of anti-TNF therapy and UST in psoriatic arthritis, specifically for enthesitis and dactylitis [68,69].

Real-life observational data support the use of UST for cutaneous EIMs in IBD patients who have failed TNF antagonists. In summary, UST appeared to be useful for different cutaneous lesions, whereas VDZ does not appear to be useful for lesions that are independent of disease activity [68].

The post hoc analysis of UNITI study [70] showed no significant resolution of EIMs for CD treated with UST compared to placebo at weeks 6 and 52; however, at the baseline, 471/941 patients had arthritis or arthralgia, of which 151 (16%, *p* < 0.0001) resolved at week 6. In the IM-UNITI analysis 129/263 patients had arthritis/arthralgia at baseline and 89 (33%, *p* < 0.0001) resolved at week 52. For erythema nodosum and iritis/uveitis the results are significant too.

### 7.4. Pregnancy

Pregnancy can modify the disease course, particularly in patients with UC where intrapartum and post-partum flare might be more frequent. On the other hand, pregnancy has minimal effect on the disease course in women with CD [71]. Hormonal influence on cytokine polarization and subsequent disease activity has been increasingly acknowledged [72]. The Toronto Consensus Statements for the Management of IBD in Pregnancy [73] and the American Gastroenterological Association’s IBD in Pregnancy Clinical Care Pathway [74] recommend maintaining medical therapy throughout pregnancy (except for methotrexate and more recently, tofacitinib) to optimize maternal-fetal outcomes.

In summary, for perianal fistulizing CD, current guidelines support the use of infliximab as the first-line biologics for inducing and maintaining fistula healing based on RCTs with a fistula-specific endpoint. ADA can be used as an alternative first line based on its similar efficacy in fistula response. However, there is insufficient evidence to recommend using VDZ or UST as first line therapy, yet they can be considered as second-line treatment in patients who had inadequate response to anti-TNF.

Regarding the elderly population, there is no consensus on the suggested sequencing among biologicals based on safety and/or efficacy, although available data show no significant differences in the safety profile of the available biologic therapies and also do not suggest a diminished clinical efficacy.

In patients with extraintestinal manifestations, anti-TNF therapy stands as first choice. In case of loss of response, UST follows as a second option.

Concerning pregnancy, so far, there is not enough evidence to suggest biological therapy sequencing, although methotrexate and tofacitinib must be avoided during this period as they have been shown to be teratogenic.

## 8. Biosimilars

In the history of biologics, the more widespread use of the biosimilars is a pivotal step. The switch and reverse switch were proved to be safe and therefore do not affect sequencing strategies.

The incidence of IBD is increasing worldwide. According to Kaplan et al. [75], developing countries are in an emergence stage, during which sporadic cases of IBD are documented. Newly industrialized countries are in the acceleration stage, during which incidence rises and prevalence is relatively low. Countries of the Western world are in the stage of compounding prevalence, during which incidence is stable, but prevalence is rising steeply. In the future the slope of the prevalence increase will level off with the transition to the prevalence equilibrium stage, which represents the opposing force between an ageing IBD population and the incidence of IBD. This implies that more and more patients will need biologic therapies; of note, not all IBD patients need biologic therapies.

The biosimilars define the way of thinking and treating patients in the last several years. In 2013, the European Medicines Agency (EMA) approved the use of the first infliximab biosimilars in IBD (remsima and inflectra). In Europe, a mandatory switch for financial reasons became accepted, and there are real-life published data on the efficacy of these treatments. One of the first countries to have a mandatory switch regulation was Hungary. Gecse et al. [76] performed a prospective study on a nationwide, multicenter, observational cohort designed to examine the efficacy, safety, and immunogenicity of CT-P13 infliximab biosimilar in the induction treatment of Crohn’s disease and ulcerative colitis. The early and the long-term data both showed comparable efficacy and safety results to the originators.

Komaki et al. [77] performed in 2017 a systematic review and meta-analysis to evaluate the efficacy and safety of biosimilars of anti-TNF agents in patients with IBD. Eleven observational studies reporting outcomes in 829 patients treated with biosimilar of IFX (CT-P13) were identified. As the main results, the pooled rates of clinical response among CD and UC at 8–14 weeks were 0.79 (95% CI = 0.65–0.88) and 0.74 (95% CI = 0.65–0.82), respectively, and at 24–30 weeks were 0.77 (95% CI = 0.63–0.86) and 0.77 (95% CI = 0.67–0.85) respectively. Adverse events were rare (CD, 0.08 (95% CI = 0.02–0.26); UC, 0.08 (95% CI = 0.03–0.17)). The pooled rates of sustained clinical response among CD and UC after switching from IFX to CT-P13 at 30–32 weeks were 0.85 (95% CI = 0.71–0.93) and 0.96 (95% CI = 0.58–1.00), respectively, and at 48–63 weeks were 0.75 (95% CI = 0.44–0.92) and 0.83 (95% CI = 0.19–0.99 respectively. Adverse events were rare (CD, 0.10, 95% CI = 0.02–0.31; UC, 0.22, 95% CI = 0.04–0.63). This meta-analysis concluded that the biosimilar CT-P13 was associated with excellent clinical efficacy and safety profile, supporting its use in IBD patients.

Ye et al. [78], in 2019, evaluated the efficacy and safety of biosimilar CT-P13 compared with originator IFX in patients with active CD. In all, 220 patients were enrolled: 111 were randomly assigned to start CT-P13 (56 to the CT-P13-CT-P13 group and 55 to the CT-P13-infliximab group) and 109 to start IFX (54 to the infliximab-infliximab group and 55 to the infliximab-CT-P13 group). CDAI-70 response rates at week 6 were similar to CT-P13 (77 [69.4%, 95% CI 59.9 to 77.8] of 111) and IFX (81 [74.3%, 95% CI 65.1 to 82.2] of 109; difference −4.9% [95% CI −16.9 to 7.3]), thereby establishing non-inferiority. At the end of the study, 147 (67%) patients experienced at least one treatment-emergent adverse event (36 [64%] in the CT-P13-CT-P13 group, 34 [62%] in the CT-P13-infliximab group, 37 [69%] in the infliximab-infliximab group, and 40 [73%] in the infliximab-CT-P13 group). In conclusion, this study showed non-inferiority of CT-P13 to IFX in patients with active CD.

Recently, Voltaire CD study [79] evaluated the efficacy and safety of the ADA’s biosimilar (BI 695501) in patients 147 patients with CD—BI 695501 (*n* = 72) and ADA (*n* = 75). At week 4, 61 (90%) of 68 patients on BI 695501 and 68 (94%) on ADA had a clinical response (adjusted RR 0.945 [90% CI 0.870–1.028]). In the safety analysis set, 45 (63%) of 72 patients on BI 695501 and 42 (56%) on ADA had an adverse event during weeks 0–24; 31 (43%) and 34 (45%) had adverse events during weeks 24–56. The most common drug-related treatment-emergent adverse events during weeks 0–24 were weight increase (three [4%] patients in the BI 695501 group) and injection-site erythema and upper respiratory tract infection (three [4%] patients for each event) in the adalimumab reference product group. Serious adverse events occurred in six (8%) patients on BI 695501 and eight (11%) on ADA between weeks 0–24, and two (3%) and nine (12%) patients between weeks 24–56. This study concluded that safety and efficacy were similar in patients with CD treated with BI 695501 or ADA. Treatment benefits were maintained in patients receiving ADA reference product who switched to BI 695501.

In summary, anti-TNF biosimilars (CT-P13 and BI 695501) demonstrated similar efficacy and safety profile compared to their originators, therefore supporting their use in IBD patients. Moreover, prescription regulation (e.g., mandatory use of biosimilars, tiering) may influence biological choice in some geographic areas.

## 9. Positioning of Biological and Small Molecule Therapies in Updated Practical Guidelines Recommendation

Current practical guidelines recommend anti-TNF as the first-line biological therapy in biologic-naïve patients with moderately to severely active luminal Crohn’s disease or ulcerative colitis. AGA guidelines recommend that ustekinumab can be used as the first-line therapy in Crohn’s disease. Vedolizumab is recommended as the first-line therapy in ulcerative colitis as recommended in AGA and ECCO guidelines. In patients with a loss of response to anti-TNF therapy, therapeutic drug monitoring (TDM) can be used for dose optimization. Switching out of class is reasonable in patients with adequate drug concentration [80]. Summary of guideline recommendations on biological therapy is summarized in Table 3.

## 10. Conclusions

Based on the landmark clinical trials, comparative randomized clinical trials and real-world studies, there is not enough evidence to suggest an optimal biological therapy sequencing (first or subsequent line). However, certain scenarios, e.g., perianal disease, elderly population, extra-intestinal manifestations, and pregnancy may influence choice of biological therapy. Similarly, prescription regulation (e.g., mandatory use of biosimilars, tiering) may influence biological choice in some geographic areas.

## Figures and Tables

**Table 1:** Summary of selected real-world studies comparing biologics in biologic-naïve patients with luminal Crohn’s disease.

Study (Year)	Biological Therapy	Nature of the Study	Studied Population	No. of Patients	Main Result
Cosnes et al. (2016) [27]	IFX vs. ADA	Single Center Retrospective Cohort	Caucasian Population	1284 (70% biologic-naïve) IFX (763) ADA (521)	Clinical response rates at 6 and 24 months: IFX 72% and 45% vs. ADA 64% and 44%, respectively.
Narula et al. (2016) [26]	IFX vs. ADA	Multicentric Retrospective Cohort	Caucasian Population	362 patients (251 IFX, 111 ADA)	At 12 months, clinical remission (IFX 50.4% vs. ADA 57.3%, *p* = 0.48) and steroid-free remission (IFX 44.3% vs. ADA 53.7%, *p* = 0.16).
Macaluso et al. (2019) [28]	IFX vs. ADA	Multicentric Retrospective Cohort	Caucasian Population	IFX (126) ADA (437)	At 12 weeks, steroid-free remission and clinical response (ADA 81.8% vs. IFX 77.6%, adjust OR: 1.23, 95% CI 0.63–2.44) At 1 year, ADA 69.2% vs. IFX 64.5 (adjust OR: 1.10, 95% CI 0.61–1.96)
Singh et al. (2018) [30]	IFX vs. ADA	Multicentric Retrospective Cohort	Caucasian Population	IFX (512) ADA (315)	No difference in CD-related hospitalization: HR 0.81 (95% CI 0.55–1.20), abdominal surgery: HR 1.24 (0.66–2.33) and Serious infections: HR 1.06 (0.26–4.21) over 2.3 year follow-up between IFX and ADA
Macaluso et al. (2021) [31]	VDZ vs. ADA	Multicentric Retrospective Cohort	Caucasian Population	585 (57% biologic-naïve, non specified in subgroups) VDZ (277) ADA (308)	A clinical response at week 12: 64.3% VDZ vs. 83.1% ADA, *p* = 0.107. At 52 weeks: 54.0% VDZ vs. 69.1% ADA (OR 0.77, 95% CI 0.45–1.31, *p* = 0.336). Endoscopic healing: 31.8% VDZ vs. 33.8%ADA, *p* = 0.85.
Bohm et al. (2020) [32]	VDZ vs. anti-TNFs (IFX, ADA, and CTZ)	Multicentric Retrospective Cohort	North American Population	VDZ (61) IFX (161), AZA/CTZ (130)	Clinical remission (HR 1.861, 95% CI 1.06–3.27) and steroid-free remission (HR 5.60, 1.47–21.37) favored VDZ over ADA/CTZ, no significantly different between VDZ and IFX. Rates of non-infectious SAEs (OR, 0.072, 0.01–0.24) but not serious infections (OR 1.18, 0.78–1.79) were significantly lower with VDZ vs. anti-TNF.
Ko et al. (2021) [33]	UST vs. VDZ vs. anti-TNF	Multicentric Retrospective Cohort	Australian Population	IFX (837) ADA (1069) VDZ (56) UST (61)	Rates of treatment persistence at 12 months, UST 80.0%, VDZ 73.5%, IFX 68.1% and ADA64.2% (*p* = 0.01)

**Table 3:** Summary of guidelines recommendations on biological therapy.

Disease	Clinical Guidelines	First-Line	Second-Line
Crohn’s disease	AGA 2021 [81]	Anti-TNF (IFX or ADA) or UST	○ FX (if ADA was the first-line use) ○ Primary anti-TNF non-response: UST or VDZ ○ Secondary anti-TNF non-response: ADA or UST or VDZ
	ECCO 2020 [82]	Anti-TNF (IFX or ADA or CTZ) Fistulizing CD: IFX	○ Anti-TNF non-response: UST or VDZ ○ Fistulizing CD: ADA (if not respond to IFX)
	Canadian 2019 [83] and Toronto 2019 [84]	Anti-TNF (IFX or ADA) Fistulizing CD: IFX or ADA	○ Anti-TNF non-response: VDZ or UST ○ Fistulizing CD: no recommendation
	AOCC and APAGE 2020 [85]	Anti TNF’s	○ No recommendation
Ulcerative colitis	AGA 2020 [86]	IFX or VDZ	○ IFX-non response: UST or Tofacitinib
	ECCO 2017 [87]	Anti-TNF (IFX, ADA) or VDZ	○ Different anti-TNF ○ Anti-TNF non-response: VDZ
	Toronto 2015 [88]	Anti-TNF	○ Primary anti-TNF non-response: VDZ ○ Secondary anti-TNF non-response: another anti-TNF or VDZ
	AOCC and APAGE 2020 [85]	Anti TNF’s	○ No recommendation

## Data Availability

The main data are given in this article. The data are available from the corresponding author upon request.
